# Therapeutic dilemmas: cognitive enhancers and risk of falling in older adults—a clinical review

**DOI:** 10.1007/s41999-023-00821-x

**Published:** 2023-07-07

**Authors:** Gabbie E. Portlock, Matthew D. Smith, Eveline P. van Poelgeest, Tomas James Welsh, on behalf of the EuGMS Task and Finish Group on FRIDs (Fall-Risk-Increasing Drugs)

**Affiliations:** 1 Bristol Medical School, University of Bristol, Bristol, UK; 2 The Research Institute for the Care of Older People (RICE), The RICE Centre Royal United Hospital, Combe Park, Bath, BA1 3NG UK; 3 Amsterdam University Medical Center, Amsterdam, The Netherlands; 4 Royal United Hospitals Bath NHS Foundation Trust, Bath, UK

**Keywords:** Cognitive enhancers, Falls, Deprescribing, Dementia

## Abstract

**Purpose:**

Cognitive enhancers are the primary pharmacological therapy prescribed to those with dementia, comprising of memantine and the acetylcholinesterase inhibitors (AChEIs). The long-term cognitive and behavioural benefits of these medications, as well as their potential contribution to falls is currently debated, with recent Delphi studies being unable to reach consensus on whether these medications should be deprescribed. In this narrative clinical review, as part of a series on deprescribing in people at risk of falls, we explore the potential falls-related side effects experienced in people taking cognitive enhancers, alongside situations where deprescribing may be appropriate.

**Methods:**

We undertook a literature search of PubMed and Google Scholar, using terms capturing falls and cognitive enhancers, as well as consulting the British National Formulary and published Summary of Medicinal Product Characteristics. These searches informed the subsequent clinical review.

**Results:**

Cognitive enhancers should be subject to regular review, including confirmation of appropriate treatment indication, and occurrence of side effects in the context of falls. AChEIs, in particular, are associated with a broad range of side effects that can contribute to increased falls risk. These include bradycardia, syncope and neuromuscular effects. Where these have been identified, deprescribing should be considered, as well as alternative treatment options. Deprescribing studies have shown mixed results, likely due to considerable methodological heterogeneity. Several suggested guidelines exist to aid deprescribing decisions, many of which are highlighted in this review.

**Conclusions:**

The use of cognitive enhancers should be regularly reviewed and decisions to deprescribe made on a case-by-case basis, considering both the risks and benefits of stopping these medications.

## Key summary points

**Aim:**

To summarize the falls-related risks of cognitive enhancing medications used to treat neurodegenerative conditions. To assist the clinician in considering the appropriateness of prescribing a cognitive enhancer in an older person at risk of falls and explore the factors that should be considered when reviewing the ongoing use of these medications.

**Findings:**

Cognitive enhancer use is associated with multiple fall-related side effects, particularly in the initial weeks of administration and up-titration. Acetylcholinesterase inhibitors and memantine differ in their fall-related side effect profiles. Cognitive enhancers can contribute to falls through precipitating cardiac, sleep, neurological (e.g. seizure), extrapyramidal, bladder and/or neuro-psychiatric side effects. Cognitive enhancers should be subject to frequent medication review, including appropriate treatment indication and occurrence of side effects. Several suggested guidelines exist to aid deprescribing decisions, however, these are based on a relatively small number of focused clinical trials.

**Message:**

An individualised approach should be taken when considering whether cognitive enhancer therapy is still appropriate in older adults at risk of falling. Clinicians should consider the plethora of risks associated with (de-) prescribing these medications.

## Introduction

Almost 700,000 individuals die annually from falling, with the vast majority of falls reported in people over the age of 65 [1]. Mortality can occur through direct injury [2], such as fracture or cerebral trauma, as well as medical complications associated with immobility and hospitalisation. The tendency to fall is associated with increased dependency, loss of autonomy, mobilisation, anxiety and depression [3–5]. In addition, falls carry a significant socio-economic cost; in the UK, falls cost the National Health Service more than £2.3 billion per year [6]. Thus, preventing falls and related injuries is imperative for maintaining the health, wellbeing and independence of older people.

An estimated 50 million people worldwide were living with dementia in 2018 and this is predicted to rise to 152 million by 2050[7]. Dementia is associated with at least double the risk of falling, alongside a significantly increased risk of head injuries and hip or arm fracture [8–10]. Furthermore, fallers with dementia are more likely to be admitted to institutional care than older people with dementia who do not fall [11, 12]. Interestingly, Ballard et al.demonstrated that older adults with dementia with Lewy bodies (DLB) were more likely to experience recurrent falls (defined as > 5 falls over a 3-month period) than those with Alzheimer’s disease (37% DLB vs 6% AD) [13]. This higher risk is likely linked to parkinsonism. Indeed, falls prevention has been identified as a top research priority by people living with Parkinson’s disease (PD) [14]. Prospective studies report that around 61% of people with PD have at least one fall in a year and 39% fall recurrently [15]. These falls are often linked to visual hallucinations, cognitive decline and hospitalisation [16].

The acetylcholinesterase inhibitors (AChEIs) donepezil, rivastigmine and galantamine and the *N*-methyl-d-aspartate (NMDA) receptor antagonist memantine are recommended in several countries for the symptomatic management of dementia due to AD and Lewy body disease (DLB and PD with related dementia; PDD). Multiple trials have demonstrated modest cognitive benefits in those prescribed these cognitive enhancers [17–21]. Exemplifying this, a recent Cochrane review reported that donepezil improved ADAS-Cog (Alzheimer’s Disease Assessment Scale–Cognitive Subscale) scores by a mean of − 2.67 points (95% CI − 3.31 to − 2.02) and MMSE (Mini Mental State Examination) scores by just 1.05 points (95% CI 0.73–1.37) [18]. Moreover, in randomised control trials (RCTs), participants treated with AChEIs for between 3 and 6 months experience small benefits in cognitive function, activities of daily living and clinician‐rated global clinical state [18–20]. Furthermore, the length of time that cognitive enhancers provide benefit to those with dementia is contested. Several systematic reviews remark that current RCTs rarely run for longer than 6 months [18, 20, 21]. Thus, it has been postulated that donepezil (and perhaps other cognitive enhancers) provides cognitive benefit for 6–9 months of treatment, which may then attenuate over the next 2 years [22].

Alongside their modest benefits on cognition and overall functioning, cognitive enhancers are associated with certain fall-related side effects, including syncope, bradycardia, sedation/sleep disorders, seizures and urinary incontinence [23–26]. Rarely and idiosyncratically, AChEIs can also worsen symptoms of parkinsonism in PDD and DLB [27]. A major challenge facing the clinician reviewing a patient with dementia, who is reporting falls, is untangling the risk of falling due to underlying disease progression versus potential fall-related side effects caused by cognitive enhancer use.

### Aims and search methodology

This narrative clinical review was informed by a literature search conducted in PubMed and Google Scholar, initially in February 2022 and updated monthly up to October 2022 (with citation and reference checking). Cognitive enhancer Summary of Product Characteristics (SmPC), personal reference libraries and the British National Formulary (BNF) were also utilized. Keywords for the searches included “falls”, “donepezil”, “galantamine”, “rivastigmine”, “memantine”, “deprescribing” and “older adults” (and appropriate variations). The selection process for clinical trial data referenced in this review included: a clinically relevant population of participants (e.g. older adults with AD); control group included; falls-related side effect reports as outcome measures and appropriate measure of associations between cognitive enhancer use and falls (e.g. risk/odds ratios). Post selection, a quality assessment of relevant trial data and review material was undertaken. Consideration was given to methodological rigour and risk of bias where relevant, although was not systematically assessed.

This review highlights factors that should be assessed and considered during review of cognitive enhancer use in older adults with dementia (and related disorders). This will aid the clinician in ensuring that cognitive enhancers are not being prescribed inappropriately, or causing side effects that may compromise the patient’s quality of life. We begin by highlighting broader topics that should be covered at cognitive enhancer review, then move into a more detailed analysis of falls risk and fall-associated side effects in those taking cognitive enhancers. This review is part of a wider series aiming to aid the clinician in clinical situations where drugs that are considered fall-risk-increasing (FRIDs) may be considered for deprescribing. We do not provide an exhaustive assessment of the literature on all cognitive enhancers and their relationship to falling, rather we focus on informing clinicians on how the most frequently used agents may precipitate fall-risk increasing side effects.

## Medication review and reconciliation

### Appropriate indication

When deciding whether to prescribe or deprescribe a cognitive enhancer, the clinician must first identify whether there is an appropriate indication to treat. In a meta-analysis conducted by Lanctôt et al., the pooled mean of global responders with AD to AChEI treatment was 9% in excess of those on placebo treatment (95% CI 6–12%) [28]. The numbers needed to treat (NNT) for 1 additional patient to benefit were 7 (95% CI 6–9) for stabilisation; 12 (95% CI 9–16) for minimal improvement and 42 (95% CI 26–114) for marked improvement [28]. This analysis exemplifies the reasonable evidence for the use of cognitive enhancers in people with AD, with other studies indicating that treatment is also appropriate for DLB or PDD [29, 30]. Whilst in people with pure vascular dementia, there is only limited evidence of clinically significant benefit [21, 31, 32]. In two recent guidelines published to aid deprescribing of cognitive enhancers, there was consensus agreement that these medications should be discontinued in people with frontotemporal dementia and other specified neurodegenerative conditions such as idiopathic PD (without dementia) [33, 34]. Similarly, evidence for the use of cognitive enhancers in people with mild cognitive impairment (MCI) was felt to be limited and thus, pharmacological treatment is not currently advised in most countries [34, 35].

### Dose and formulation

All cognitive enhancers should be initiated at the lowest possible starting dose and slowly titrated up to maintenance dose. If the patient is reporting troublesome side effects (particularly cardiac), then consider either stopping the drug or down titration to a lower dose [33]. Adverse effects (AEs) are often mild and transient when the cognitive enhancer is titrated up slowly, over the course of 1–3 months [36]. AEs tend to occur during initial dose-escalation, so close monitoring of patients starting cognitive enhancers should be performed, particularly in the first 2 months [24, 25].

Dose adjustment and particularly cautious up-titration should be performed in those with renal or hepatic impairment. For example, the BNF stipulates that patients should not be treated with memantine if their estimated glomerular filtration rate (eGFR) is < 5 mL/min/1.73 m^2^ [24]. Furthermore, the dose of memantine should be halved if eGFR is 30–49 mL/min/1.73 m^2^ [24]. In the case of mild–moderate hepatic impairment, titration of galantamine tablets over 5 weeks up to a maximum daily dose of 16 mg is advised, with close monitoring of individual dose tolerability [26]. All cognitive enhancers should be avoided in those with severe hepatic impairment [23–26].

The most reported side effects for AChEIs are gastrointestinal (GI); some sources suggest that taking AChEIs with food reduces the likelihood of GI symptoms [37, 38]. Although the oral administration route may be easiest for the patient, if this is compromised or GI side effects are severe, then rivastigmine is also available in once daily transdermal patch formulation [39]. The GI side effect profile for these patches is reduced, but clinicians should check for history of skin conditions such as eczema, as the patches can cause skin sensitivity, rashes and redness where they are placed [40]. Clinicians should be particularly aware of overdose symptoms with rivastigmine patches—this occurs when patients or their carers inadvertently apply more than one patch at a time [41, 42].

### Cautions and contraindications

A check for any previously missed contra-indications is warranted in any medication review. In the case of the donepezil, caution should be exercised with co-existing asthma, chronic obstructive pulmonary disease (COPD), previous peptic ulcers and cardiac conduction problems such as sick sinus syndrome [25]. In the case of rivastigmine and galantamine, the clinician should additionally check for history of seizures, risks of bladder or GI obstruction and other cardiac diseases such as congestive heart failure or angina [23, 43]. In the case of memantine, the main caution is in people with previous seizure disorder [44], likely borne more out of its theorised activity on the NMDA receptor than empirical data. Indeed, clinical trial data indicate no increased risk of seizure (compared with AChEI treatment) and memantine has even been assessed to improve cognition in patients with epilepsy [45, 46]. Moreover, navigating the above cautions requires a careful balance of risk versus benefit, in a population that has increasing co-morbidities.

Notably, if the clinical context has changed significantly over time, for example, where a patient is acutely unwell with delirium, severe sepsis or developed a new cardiac arrhythmia, then medication such as cognitive enhancers may need to be suspended or the dose adjusted.

### Drug–drug interactions

A full medication history should be taken at each review, considering currently co-prescribed drugs and alcohol use. Many older adults that are candidates for cognitive enhancer prescription also have multiple co-morbidities and consequent polypharmacy. This significantly increases the risk of drug–drug interactions, particularly noted for galantamine, as its main route of metabolism is through the cytochrome P450 enzymes CYP2D6 and CYP3A4 [47]. Many other drugs are metabolised by the CYP enzymes, which can lead to galantamine lingering in the body for longer time periods. For example, the bioavailability of galantamine has been reported to increase by 40% when co-administered with paroxetine and 30% with co-administration of ketoconazole [48]. Rivastigmine would be a good alternative to galantamine in those who take many other medications, due its non-cytochrome P450 metabolism [47]. Regarding the AChEIs, co-prescription of negatively chronotropic cardiac drugs (beta-blockers and digoxin) should trigger close monitoring of heart rate, due to the potential for these combinations to cause significant bradycardia [49]. Despite this potential interaction, the propensity of co-prescription of beta blockers and AChEIs has been suggested not to cause increased fall-related injury in people living in nursing homes [50].

Memantine may interact with dopaminergic medication taken by people with PDD (based on mostly animal model data), leading to an inappropriate increase in central dopamine [44, 51]. Dopaminergic agents alone can worsen or precipitate neuropsychiatric symptoms, and thus if prescribed alongside memantine, adjusting the dose of dopaminergic medication should be considered. A serious interaction between amantadine and memantine has been documented, likely due to their similar mechanisms of action [44, 52, 53]. Despite this, recent Adverse Drug Event Reports suggest that such an interaction may not be clinically significant, although this is based on a small number of cases [54].

Older adults may be concurrently prescribed anti-cholinergic medication to treat bladder problems, such as solifenacin or oxybutynin [55]. Extensive literature documents concern about anticholinergic medications and increased risk of cognitive impairment [56, 57]. The rationale for prescribing medications with anticholinergic effects should be carefully examined in those with cognitive impairment, who have also been prescribed an AChEI [58]. Anticholinergic drugs and AChEIs have antagonistic mechanisms of action and thus, could cancel out each other’s therapeutic benefits whilst adding further side-effects. Moreover, discontinuation of anticholinergic medication may worsen urinary symptoms and indirectly increase the risk of falls, whilst introduction of an AChEI may also contribute to these symptoms worsening [56]. Here, it is key to consider whether alternative medications (such as mirabegron or memantine) with less activity at cholinergic receptors would provide the patient with symptomatic relief [59]. It is also important to assess for the presence of drugs with unintentional cholinergic burden, a good example being amitriptyline [60]. These risks can be assessed using online scoring tools such as the ACB (anticholinergic burden score: https://www.acbcalc.com/).

### Non-pharmacological treatments

Cognitive Stimulation Therapy (CST) is an evidence based psychological intervention for people with mild to moderate dementia, which results in similar cognitive improvements to memory enhancing medications [61, 62]. Group CST is the only non-pharmacological intervention for dementia recommended in the UK and is now established across the globe [63]. This intervention should be offered to all patients with mild to moderate dementia, regardless of subtype or prescription of cognitive enhancers, if the clinician and patient feel it would be helpful [64]. CST provides a good alternative for those in whom cognitive enhancers are contra-indicated or deemed inappropriate.

## Falls and fall-related adverse events

Several falls-risk related side effects are associated with cognitive enhancer use (prevalence displayed in Table 1). For the AChEIs, these can be grouped into cardiac, sleep-related, neurological (e.g. seizure), extrapyramidal, bladder and neuro-psychiatric effects. For memantine, potential AEs include balance impairment, dizziness, confusion, hallucination and seizure [44]. Notably, the only cognitive enhancer in which the SmPC mentions falls specifically as a common side effect is galantamine. This could be because it has a dual mechanism of action as both an AChEI and a direct nicotinic receptor agonist [41], although may simply be due to variation in trial design and vigilance. With all AChEIs potentiating peripheral nicotinic receptors through enhanced acetylcholine signalling at the neuromuscular junction, this can cause muscle cramps, weakness and extrapyramidal symptoms which could contribute to increased falls incidence [65].

**Table 1:** Prevalence of fall-related side effects for cognitive enhancers

Cognitive enhancer	Cardiac conduction disorder	Arrhythmia	Syncope	Seizure	Sedation /sleep disturbance	Neuro-psychiatric e.g. altered behaviour, mood, hallucinations	Dizziness	Urinary incontinence	Extrapyramidal symptoms including tremor	Fall
Donepezil	+	++	+++	++	+++	+++	+++	+++	+	+++
Rivastigmine	++	+++	+++	+	+++	+++	+++	+++	+++	++++(PDD)
Galantamine	++	+++	+++	++	+++	+++	+++	No data	+++	+++
Memantine	N/A	N/A	No data	+	+++	++	+++	N/A	N/A	No data

+: < 1/1000

++:1/100–1/1000

+++:1/10–1/100

++++: > 1/10

Source: Cognitive enhancer SmPCs[53]

Kim et al. provide a comprehensive meta-analysis on falls and related AEs reported for AChEI use in dementia cohorts. Pooled odds ratios (ORs) for falls (OR = 0.88, *p* = 0.14) and fracture (OR = 1.39, *p* = 0.29) whilst taking AChEIs were not significantly different to placebo treatment, suggesting no benefit but also no increase in risk [66]. These results remained non-significant when subgroup analysis accounting for disease severity was included. However, a significantly increased risk of syncope was seen (OR = 1.53, *p* = 0.04) in those using AChEIs, alongside bradycardia being a frequently reported AE [66].

Kim et al. also quantified memantine’s association with risk of falls and related events in dementia cohorts. Memantine was associated with fewer fractures (OR = 0.21, 95% CI 0.05–0.85) and did not significantly influence falls risk (OR = 0.92, 95% CI 0.72–1.18) or fall-related events (syncope or accidental injury; OR = 1.04, 95% CI 0.35–3.04 and OR = 0.80, 95% CI 0.56–1.12) [66]. This agrees with a Cochrane review of memantine use for dementia, which reported high-certainty evidence of no difference between memantine and placebo for falls (RR = 0.98, 95% CI 0.84–1.13) [21]. However, a possible consideration for understanding this finding is that those taking memantine may be at a later disease stage, and thus may be more sedentary, minimising their exposure to walking and therefore risk of falling. Additionally, the effect of drop out on more severely affected individuals may skew the results of clinical trials.

The above are limitations in most of the clinical trial data currently available to quantify falls and related side effects across the dementia population [67]. Moreover, there is a dearth of RCTs that reliably document falls in older adults with dementia (particularly higher dependency individuals). This may simply be because minimal falls occur within the generally short clinical trial timescales and are, therefore, not deemed worthy of inclusion in final analyses. However, it could also be due to inconsistencies defining and recording falls in RCTs, making it challenging to extract this data and extrapolate it across the whole dementia population.

### Cardiovascular side effects

AChEIs increase vagal tone, potentially affecting the sinoatrial node and dynamic control of heart rate [68]. Thus, caution is required when prescribing AChEIs to individuals who already have bradycardia or cardiac conduction abnormalities such as sick sinus syndrome. Patients and their carers should be warned about the risk of syncope and presyncope due to low heart rate when AChEIs are prescribed. Moreover, Birks’ Cochrane review identified syncope as a side effect associated with all AChEIs (OR = 1.9, 95% CI 1.09–3.33) [69] and, as described earlier, the risk of bradyarrhythmia is higher if other rate control medications are co-prescribed. Due to the increased risk of bradycardia in patients with a resting heart rate of less than 60 bpm [70], cognitive enhancers should be used with caution (particularly those with frailty), including review of heart rate at titration or consideration of memantine as an alternative. An ECG should be considered to look for evidence of heart block, particularly if there is a history of unexplained syncope or cardiac disease [68]. In an individual who has experienced unexplained syncope, AChEIs should be avoided and if syncope or presyncope develops after AChEIs have been started, the medication should be discontinued. Finally, patients should be monitored for emergence of orthostatic hypotension when cognitive enhancers are introduced. This can precipitate from the bradycardic effects of AChEIs, alongside increased dehydration and autonomic dysfunction from concomitant alpha-synucleinopathy (PDD or DLB) [71].

### Sleep disturbance and sedation side effects

Sleep disorders such as vivid dreams and insomnia are common side effects in older adults treated with AChEIs [37, 70]. Vivid dreams are reported in patients on donepezil in particular, likely owing to current advice to take it at night-time in order to sleep through any GI or dizziness side effects [72]. Abnormal dreams were reported by up to 1 in 10 participants on AChEI treatment in 1 study (OR = 5.38, 95% CI 1.34–21.55) and insomnia in 1 in 12 participants (OR = 1.49, 95% CI 1.12–2.0) [37]. Poor quality sleep at night can lead to day-time somnolence, which in turn can increase the risk of falls. If patients experience insomnia, changing to morning administration has been suggested [72].

Memantine is less likely to cause sleeping difficulties, with McShane’s Cochrane review reporting no increased risk (risk ratio (RR) = 0.93, 95% CI 0.73–1.2), although the evidence was graded as low[21]. Further, memantine has been reported to decrease REM sleep behaviour disorder (RBD) symptoms in patients with DLB and PDD [73]. The mechanism through which this occurs is still unknown, however, it was posited that memantine’s neuroprotective properties, alongside possible potentiation of dopamine in the prefrontal cortex contributed to the reduction RBD symptoms [73].

### Neuromuscular side effects: cramps and weakness

Muscle cramps are a very common side effect of the AChEIs (OR = 13.32, 95% CI 1.71–103.7) [69]. These can be debilitating, lead to a reduction in physical activity and a consequent contribution to increased falls risk. Furthermore, fatigue is also a common side effect of the AChEIs (OR = 4.39, 95% CI 1.21–15.85) [69]. This may relate to loss of appetite and weight loss, which are often observed in patients taking cognitive enhancers (OR = 2.99, 95% CI 1.89–4.75) [69]. Accordingly, weight changes should be monitored closely when patients are first started on AChEIs, and weight should be measured at every follow-up appointment for the duration of treatment. There is some evidence (from a single trial) that weight loss was less common in people taking donepezil than oral rivastigmine (OR = 0.28, 95% CI 0.13–0.61), which may indicate it as a preferred option in patients with low baseline BMIs [69, 74]. Notably, a reduction in physical activity, fatigue and weight loss are all contributing factors to increased falls risk [75, 76], although whether this is causative or simply an association is unclear.

### Other neurological side effects: seizure and dizziness

Both the AChEIs and memantine are associated with a risk of dizziness: OR 1.99 (95% CI 1.59–2.30) and RR 1.59 (95% CI 1.28–1.99), respectively [21, 69]. The AChEIs are also associated with a risk of vertigo (OR 3.95, 95% CI 1.08–14.46) [69]. ‘Dizziness’ may be considered a nonspecific term for events that include light-headedness, palpitations, neurological gait dysfunction and true vertigo, which is important for the clinician to explore in more detail.

AChEIs can increase the risk of seizures (a rare but significant side effect), although it should also be noted that AD itself increases the risk of seizure disorder [77, 78]. Due to these risks and lack of RCT evidence to the contrary, cognitive enhancers should be trialled with caution and closer clinical monitoring in people with known seizure disorders [23–26]. Cognitive enhancers should only be introduced in people with seizure disorders after a discussion about the potential risks, including notifying the patient that anticonvulsants can have a negative effect on cognition, even in those without dementia [79].

### Neuropsychiatric side effects

Dementia can precipitate fall-risk increasing neuropsychiatric symptoms, such as hallucinations, delusions, aggression, agitation and wandering [80]. However, increasing central acetylcholine levels with cognitive enhancers may perpetuate or even precipitate these symptoms in people taking AChEIs [81]. Less commonly, neuropsychiatric side effects have been associated with memantine [24]. A challenge facing the clinician is untangling whether patients have developed neuropsychiatric symptoms, because they are at a more advanced disease stage, or whether their medication has contributed to these symptoms. Conversely, cognitive enhancers can also have a beneficial effect on neuropsychiatric symptoms, particularly the hallucinations that can occur in PD, reiterating how each case should be considered on an individual basis [82–84].

### Extrapyramidal side effects

AChEIs can worsen parkinsonism when given for DLB or PDD and can be linked to troublesome postural difficulties including Pisa syndrome, where problematic unilateral postural leaning is seen on walking and in some cases resting positions [85]. Tremor can be induced or worsened by cognitive enhancers (OR 6.82, 95% CI 1.99–23.37), with galantamine conferring higher risk compared to donepezil in a single study of each [43, 69]. Patients with PDD or DLB should be counselled to monitor these symptoms, particularly during dose titrations.

### Bladder-related side effects

Urinary symptoms are relatively common side effects of AChEIs, generally presenting as urinary urgency and incontinence [86]. In individuals with pre-existing urinary symptoms, caution should be taken and symptoms monitored for worsening. Monitoring should also be considered in those without a pre-existing history, as it is not uncommon for these symptoms to go unmentioned and attributed to age.

## Discontinuing cognitive enhancers

As has been discussed, there are a multitude of factors which might prompt a cognitive enhancer to be deprescribed, of particular importance in the context of falls. The cognitive enhancers are associated with several falls risk associated side effects and the occurrence of these side effects should prompt consideration of deprescribing. Likewise, the presence of other intolerable side effects should prompt a deprescribing discussion. Several consensus groups have suggested deprescribing a cognitive enhancer if no clinical benefit on cognition or behaviour is observed after 6–12 months of treatment, there has been significant decline in cognition and function despite treatment, or the patient is reaching the end of their life [33]. However, individualised treatment is key—the course of dementia is notoriously variable and what may be appropriate for one individual may be completely inappropriate for another.

Discontinuing cognitive enhancers can lead to a worsening in cognitive function—a significant concern for both patients and clinicians [87, 88]. This risk must be balanced against factors which prompt deprescribing. Guidance on change in condition post deprescribing is given in the Reeve et al. guidelines for deprescribing cognitive enhancers [33]. Table 2 in the Reeve et al. guideline describes the symptoms that may occur for up to 3 months post-deprescribing, alongside the action to be taken by family, nurses and/or care staff.

In addition, the presence of significant neuropsychiatric symptoms may incline the clinician to continue a cognitive enhancer; worsening neuropsychiatric symptoms are associated with increased falls risk in AD [89, 90] and discontinuing cognitive enhancers can worsen such symptoms. Where a decision to stop a cognitive enhancer has been made, the dose should be reduced gradually to avoid a withdrawal syndrome (typically involving neuropsychiatric symptoms like hallucinosis and delusions [91, 92]). Relevant guidelines recommend that cognitive enhancers be reduced slowly and with close observation [33, 91, 93], for example reducing by 50% every 4 weeks until the medication can be removed at its lowest dose. If significant cognitive deterioration does occur post cognitive enhancer withdrawal, re-introducing the relevant medication at the lowest therapeutic dose could be considered. Crucially, a deprescribing decision should consider the patient’s preferences or previously expressed wishes. It should also be a collaborative decision involving patient, family, carers and clinicians.

Regardless of deprescribing decisions, other modifiable falls risk factors for those with dementia should be addressed. The recently published World Guidelines for Fall Prevention advise that older adults identified as being at high risk of falling should be offered a comprehensive falls risk assessment to guide future intervention [71]. More specifically, Table 4 in World Guidelines for Fall Prevention gives detailed information on the domains relating to falls that should be assessed, including mobility, sensory function, cognitive function, disease and medication history. These guidelines then provide descriptions of interventions that should be introduced to subgroups of fallers, including community dwelling older adults, those in hospital, care homes and older adults with specific conditions such as PD, stroke and dementia [71]. Example recommendations for those with MCI and dementia include exercise programmes such as Tai Chi, education on improving diet/nutrition and reducing environmental risk factors with the help of the patient, their family and carers.

### Deprescribing cognitive enhancers and falls: trial data

Niznik et al*.* specifically assessed the effect of deprescribing cognitive enhancers on falls [94]. This was a retrospective assessment of US Medicare claim data, assessing the association between deprescribing AChEIs and the risk of adverse events including: hospitalizations, emergency department visits, mortality, falls and fractures. Deprescribing AChEIs was associated with a reduced likelihood of serious falls or fractures in both adjusted and unadjusted models (OR = 0.59, 95% CI 0.52–0.66, *p* = < 0.001 and adjusted OR = 0.64, 95% CI 0.56–0.73, *p* = < 0.001)[94]. However, although the adjusted model considered numerous confounding variables including co-morbidity and activities of daily living indices, dementia severity, specifically, was not included. The authors of this study postulate that deprescribing may lead to benefit in this cohort with severe dementia, whilst also acknowledging the limitations in their study.

In a more recent study, Moo et al*.* conducted an RCT of AChEI discontinuation in veterans with multiple dementia subtypes[95]. However, recruitment was very challenging resulting in the study being significantly underpowered. The authors highlighted the main reasons that people did not want to participate in this study as: no interest in the research topic, did not want to make medication changes, unable to provide time/effort needed for the study, had already discontinued the medication or had been advised by their clinician to keep taking it [95]. Further research is needed on deprescribing cognitive enhancers in general, as well as in comparison between dementia subtypes.

### Deprescribing cognitive enhancers guidelines and decision-aids

Several guidelines have been produced to aid decision-making in deprescribing cognitive enhancers[33, 34, 96]. Guidance in this area is, however, plagued by a scarcity of robust evidence on deprescribing, hampered by the heterogeneity in symptoms and underlying pathology presented in people with dementia. A systematic review from Renn et al*.* explored many guidelines on (de)prescribing AChEIs and highlighted the lack of consensus amongst these decision-aids[96]. Falls as a perceived side effect of treatment were commonly used as a rationale for discontinuation of cognitive enhancers[96]. However, there was minimal discussion on how discontinuing this class of medication would specifically prevent further falls.

The leading deprescribing guidance for cognitive enhancers has been produced via consensus approach by groups in Australia and Canada[33, 34]. Reflecting the heterogeneous nature of the population, a common theme includes comprehensive and individualised assessment of the patient. However, these guidelines provide a helpful framework to guide the clinician considering deprescribing (see Fig. 3 decision chart for more detail [33]). Additional material for patients to better inform shared decision-making is available at www.deprescribing.org.

## Discussion

Making assessments on the benefits or harms of cognitive enhancer therapy in the context of falls is a challenging task. Much of the data looking at links between AChEIs, memantine and falls is derived from RCT evidence where the participants are not representative of the clinical population. This leads to considerable uncertainty when interpreting the data. Additionally, whilst trials to prove initial drug efficacy operate with strictly defined inclusion criteria, often involving early disease stages, deprescribing studies frequently involve those at later disease stages. The extent of disease severity (as well as non-neurological co-morbidity) can have a significant effect on the rate of falls. Moreover, those who in the latest stages of dementia and PD experience a paradoxical decrease in falls risk as they become bed bound, are those most likely to be considered to benefit from deprescribing. Consequently, those that have their cognitive enhancers deprescribed may appear to experience less falls when they merely embark on less risky ambulation. In the absence of conclusive evidence, it becomes more important to individualise the decision-making process, guided by patient preference.

## Conclusions

Some advice can be offered to the clinician in a dilemma, faced with an older person prescribed a cognitive enhancer who is falling:

- *Shared decision-making.* Honour patient preferences, involve the patient, their family and caregivers. Individualise the treatment.
- *Confirm the diagnosis.* If the underlying condition is **not** Alzheimer’s disease dementia, dementia with Lewy bodies, Parkinson’s disease dementia, mixed dementia, or vascular dementia deprescribe the cognitive enhancer. If the underlying condition is pure vascular dementia, consider the more limited evidence of cognitive/functional benefit and consider deprescribing on an individual basis.
- *Consider contraindications.* Are there any co-morbidities or medications which may be problematic e.g. asthma and AChEIs? Consider deprescribing if a clear contraindication is present.
- *For patients who do have an indication for treatment and who have been taking cognitive enhancers for over 1 year:* if there has been no clinically meaningful benefit, progressive deterioration in cognition despite treatment and no psychiatric symptoms, deprescribing should be considered.
- *Assess side effects.* AChEIs and memantine are associated with AEs which may lead to falls, such as dizziness, bradycardia and syncope. Clinicians should look closely for the presence of AEs and review the ongoing need for the medication accordingly. Unexplained syncope in a patient taking an AChEI should prompt review of the medication.
- *Post deprescribing monitoring phase.* If AChEIs and/or memantine are deprescribed, then this should be done gradually, and patients should be monitored for withdrawal symptoms. Treatment can be restarted if there is meaningful deterioration in cognitive function.

### Future perspectives

Future research examining the risks of falls with cognitive enhancers should be developed in such a way as to avoid the risks of significant confounding. Of relevance, studies are currently underway assessing whether enhancement of the cholinergic system using rivastigmine patches may even improve the risk of falls in PD[97]. Outcomes should be clinically meaningful, alongside improving patient safety and quality of life. Cognition, falls, and cognitive enhancers have a complex interaction—the risks and benefits change as the underlying disease progresses and patient goals and symptoms vary. Research into enhancing shared-decision-making in an inherently uncertain situation such as this is to be welcomed.

## Data Availability

No datasets were generated for this clinical review.

## References

[1] World Health Organisation (WHO). Falls—fact sheet 2021. https://www.who.int/news-room/fact-sheets/detail/falls. Accessed 11 Dec 2022

[2] Dionyssiotis Y (2012). Analyzing the problem of falls among older people. Int J Gen Med.

[3] Murphy J, Isaacs B (1982). The post-fall syndrome. Gerontology.

[4] Schoene D, Heller C, Aung YN, Sieber CC, Kemmler W, Freiberger E (2019). A systematic review on the influence of fear of falling on quality of life in older people: is there a role for falls?. Clin Interv Aging.

[5] Payette MC, Bélanger C, Léveillé V, Grenier S (2016). Fall-related psychological concerns and anxiety among community-dwelling older adults: systematic review and meta-analysis. PLoS ONE.

[6] National Institute for Health and Care Excellence (NICE) (2013) Falls in older people: assessing risk and prevention. Clinical guideline [CG161]. https://www.nice.org.uk/guidance/CG161/chapter/introduction. Accessed 25 Nov 2022 31869045

[7] Patterson C, Alzheimer’s Disease International (2018). World Alzheimer report.

[8] Shaw FE, Kenny RA (1998). Can falls in patients with dementia be prevented?. Age Ageing.

[9] Kallin K, Gustafson Y, Sandman PO, Karlsson S (2004). Drugs and falls in older people in geriatric care settings. Aging Clin Exp Res.

[10] Muir SW, Gopaul K, Montero Odasso MM (2012). The role of cognitive impairment in fall risk among older adults: a systematic review and meta-analysis. Age Ageing.

[11] Morris JC, Rubin EH, Morris EJ, Mandep SA (1987). Senile dementia of the Alzheimer’s type: an important risk factor for serious falls. J Gerontol.

[12] Shaw FE (2007). Prevention of falls in older people with dementia. J Neural Transm.

[13] Ballard CG, Shaw F, Lowery K, McKeith I, Kenny R (1999). The prevalence, assessment and associations of falls in dementia with Lewy bodies and Alzheimer’s disease. Dement Geriatr Cogn Disord.

[14] Deane KHO, Flaherty H, Daley DJ, Pascoe R, Penhale B, Clarke CE (2015). Priority setting partnership to identify the top 10 research priorities for the management of Parkinson’s disease. BMJ Open.

[15] Allen NE, Schwarzel AK, Canning CG (2013). Recurrent falls in Parkinson’s disease: a systematic review. Parkinsons Dis.

[16] Yarnall A, Rochester L, Burn DJ (2011). The interplay of cholinergic function, attention, and falls in Parkinson’s disease. Mov Disord.

[17] National Institute for Health and Care Excellence (NICE) (2018) Dementia: assessment, management and support for people living with dementia and their carers. NICE guideline [NG97]. https://www.nice.org.uk/guidance/ng97. Acessed 11 Dec 2022 30011160

[18] Birks JS, Harvey RJ (2018). Donepezil for dementia due to Alzheimer’s disease. Cochrane Database Syst Rev.

[19] Birks JS, Chong LY, Grimley EJ (2015). Rivastigmine for Alzheimer’s disease. Cochrane Database Syst Rev.

[20] Olin JT, Schneider L (2002). Galantamine for Alzheimer’s disease. Cochrane Database Syst Rev.

[21] McShane R, Westby MJ, Roberts E, Minakaran N, Schneider L, Farrimond LE (2019). Memantine for dementia. Cochrane Database Syst Rev.

[22] Seltzer B (2005). Donepezil in the treatment of dementia. Expert Opin Drug Metab Toxicol.

[23] National Institute for Health and Care Excellence (NICE) (2018) Rivastigmine. British National Formulary. https://bnf.nice.org.uk/drugs/rivastigmine/. Accessed 30 May 2022

[24] National Institute for Health and Care Excellence (NICE) (2018) Memantine hydrochloride. British National Formulary (BNF). https://bnf.nice.org.uk/drugs/memantine-hydrochloride/. Accessed 18 Jan 2023

[25] National Institute for Health and Care Excellence (NICE) (2018) Donepezil hydrochloride. British National Formulary (BNF). https://bnf.nice.org.uk/drugs/donepezil-hydrochloride/. Accessed 18 Jan 2023

[26] National Institute for Health and Care Excellence (NICE) (2018) Galantamine. British National Formulary (BNF). https://bnf.nice.org.uk/drugs/galantamine/. Accessed 18 Jan 2023

[27] Pagano G, Rengo G, Pasqualetti G, Femminella GD, Monzani F, Ferrara N (2015). Cholinesterase inhibitors for Parkinson’s disease: a systematic review and meta-analysis. J Neurol Neurosurg Psychiatry.

[28] Lanctôt KL, Herrmann N, Yau KK, Khan LR, Liu BA, LouLou MM (2003). Efficacy and safety of cholinesterase inhibitors in Alzheimer’s disease: a meta-analysis. CMAJ Can Med Assoc J.

[29] Rolinski M, Fox C, Maidment I, Mcshane R (2012). Cholinesterase inhibitors for dementia with Lewy bodies, Parkinson’s disease dementia and cognitive impairment in Parkinson’s disease. Cochrane Database of Syst Rev.

[30] Aarsland D, Mosimann UP, McKeith IG (2004). Role of cholinesterase inhibitors in Parkinson’s disease and dementia with Lewy bodies. J Geriatr Psychiatry Neurol.

[31] O’Brien JT, Holmes C, Jones M, Jones R, Livingston G, McKeith I (2017). Clinical practice with anti-dementia drugs: a revised (third) consensus statement from the British Association for Psychopharmacology. J Psychopharmacol.

[32] Battle CE, Abdul-Rahim AH, Shenkin SD, Hewitt J, Quinn TJ (2021). Cholinesterase inhibitors for vascular dementia and other vascular cognitive impairments: a network meta-analysis. Cochrane Database Syst Rev.

[33] Reeve E, Farrell B, Thompson W, Herrmann N, Sketris I, Magin PJ (2019). Deprescribing cholinesterase inhibitors and memantine in dementia: guideline summary. Med J Aust.

[34] Ismail Z, Black SE, Camicioli R, Chertkow H, Herrmann N, Laforce R (2020). Recommendations of the 5th Canadian Consensus Conference on the diagnosis and treatment of dementia. Alzheimer’s Dement.

[35] Russ TC, Morling JR (2012). Cholinesterase inhibitors for mild cognitive impairment. Cochrane Database Syst Rev.

[36] Yiannopoulou KG, Papageorgiou SG (2020). Current and future treatments in alzheimer disease: an update. J Cent Nerv Syst Dis.

[37] Buckley JS, Salpeter SR (2015). A risk-benefit assessment of dementia medications: systematic review of the evidence. Drugs Aging.

[38] Desai AK, Grossberg GT (2005). Rivastigmine for Alzheimer’s disease. Expert Rev Neurother.

[39] Sadowsky CH, Micca JL, Grossberg GT, Velting DM (2014). Rivastigmine from capsules to patch: therapeutic advances in the management of Alzheimer’s disease and Parkinson’s disease dementia. Prim Care Companion CNS Disord.

[40] Greenspoon J, Herrmann N, Adam DN (2011). Transdermal rivastigmine: management of cutaneous adverse events and review of the literature. CNS Drugs.

[41] Colovic MB, Krstic DZ, Lazarevic-Pasti TD, Bondzic AM, Vasic VM (2013). Acetylcholinesterase inhibitors: pharmacology and toxicology. Curr Neuropharmacol.

[42] Pope C, Karanth S, Liu J (2005). Pharmacology and toxicology of cholinesterase inhibitors: uses and misuses of a common mechanism of action. Environ Toxicol Pharmacol.

[43] National Institute for Health and Care Excellence (NICE) (2018) Galantamine|hepatic impairment. British National Formulary (BNF). https://bnf.nice.org.uk/drugs/galantamine/#hepatic-impairment. Accessed 11 Dec 2022

[44] National Institute for Health and Care Excellence (NICE) (2018) Memantine hydrochloride|renal impairment. British National Formulary (BNF). https://bnf.nice.org.uk/drugs/memantine-hydrochloride/#renal-impairment. Accessed 11 Dec 2022

[45] Kumlien E, Lundberg PO (2010). Seizure risk associated with neuroactive drugs: data from the WHO adverse drug reactions database. Seizure.

[46] Marimuthu P, Varadarajan S, Krishnan M, Shanmugam S, Kunjuraman GR, Ravinder J (2016). Evaluating the efficacy of memantine on improving cognitive functions in epileptic patients receiving anti-epileptic drugs: a double-blind placebo-controlled clinical trial (phase IIIb pilot study). Ann Indian Acad Neurol.

[47] Bentué-Ferrer D, Tribut O, Polard E, Allain H (2003). Clinically significant drug interactions with cholinesterase inhibitors: a guide for neurologists. CNS Drugs.

[48] Scott LJ, Goa KL (2000). Galantamine: a review of its use in Alzheimer’s disease. Drugs.

[49] Watt JA, Campitelli MPHMA, Maxwell CJ, Guan J, Maclagan LC, Gomes T (2020). Fall-related hospitalizations in nursing home residents co-prescribed a cholinesterase inhibitor and beta-blocker. J Am Geriatr Soc.

[50] Elliott T, Eckmann L, Moga DC (2021). Case report: the complexities of managing medications and the importance of deprescribing anticholinergics in older adults. Front Pharmacol.

[51] Parsons C, Rammes G, Danysz W (2008). Pharmacodynamics of memantine: an update. Curr Neuropharmacol.

[52] Kornhuber J, Weller M, Schoppmeyer K (1994). Amantadine and memantine are NMDA receptor antagonists with neuroprotective properties. J Neural Transm.

[53] Electronic Medicines Compendium (EMC) (2022) https://www.medicines.org.uk/emc#gref. Accessed 30 Jan 2023

[54] Sato K, Mano T, Iwata A, Toda T (2021). Safety of memantine in combination with potentially interactive drugs in the real world: a pharmacovigilance study using the japanese adverse drug event report (JADER) database. J Alzheimers Dis.

[55] Staskin DR (2005). Overactive bladder in the elderly: a guide to pharmacological management. Drugs Aging.

[56] Gromek KR, Thorpe CT, Aspinall SL, Hanson LC, Niznik JD (2022). Anticholinergic co-prescribing in nursing home residents using cholinesterase inhibitors: potential deprescribing cascade. J Am Geriatr Soc.

[57] Doherty AS, Shahid F, Moriarty F, Boland F, Clyne B, Dreischulte T (2022). Prescribing cascades in community-dwelling adults: a systematic review. Pharmacol Res Perspect.

[58] Gill SS, Mamdani M, Naglie G, Streiner DL, Bronskill SE, Kopp A (2005). A prescribing cascade involving cholinesterase inhibitors and anticholinergic drugs. Arch Intern Med.

[59] Wagg A, Nitti VW, Kelleher C, Castro-Diaz D, Siddiqui E, Berner T (2016). Oral pharmacotherapy for overactive bladder in older patients: mirabegron as a potential alternative to antimuscarinics. Curr Med Res Opin.

[60] Brueckle MS, Thomas ET, Seide SE, Pilz M, Gonzalez-Gonzalez AI, Nguyen TS (2020). Adverse drug reactions associated with amitriptyline—protocol for a systematic multiple-indication review and meta-analysis. Syst Rev.

[61] Woods B, Thorgrimsen L, Spector A, Royan L, Orrell M (2007). Improved quality of life and cognitive stimulation therapy in dementia. Aging Ment Health.

[62] Spector A, Thorgrimsen L, Woods B, Royan L, Davies S, Butterworth M (2003). Efficacy of an evidence-based cognitive stimulation therapy programme for people with dementia: randomised controlled trial. Br J Psychiatry.

[63] Spector A, Orrell M, Woods B, Fisher E. Cognitive stimulation therapy 2023. http://www.cstdementia.com/. Accessed 28 Dec 2022

[64] Aguirre E, Hoare Z, Streater A, Spector A, Woods B, Hoe J (2013). Cognitive stimulation therapy (CST) for people with dementia—who benefits most?. Int J Geriatr Psychiatry.

[65] Adeyinka A, Kondamudi NP (2022) Cholinergic crisis. StatPearls. 10.1007/978-3-211-79280-3_237

[66] Kim DH, Brown RT, Ding EL, Kiel DP, Berry SD (2011). Dementia medications and risk of falls, syncope, and related adverse events: meta-analysis of randomized controlled trials. J Am Geriatr Soc.

[67] Freiberger E, de Vreede P (2011). Falls recall-limitations of the most used inclusion criteria. Eur Rev Aging Phys Act.

[68] Ruangritchankul S, Chantharit P, Srisuma S, Gray LC (2021). Adverse drug reactions of acetylcholinesterase inhibitors in older people living with dementia: a comprehensive literature review. Ther Clin Risk Manag.

[69] Birks J (2006). Cholinesterase inhibitors for Alzheimer’s disease. Cochrane Database Syst Rev.

[70] Kavirajan H, Schneider LS (2007). Efficacy and adverse effects of cholinesterase inhibitors and memantine in vascular dementia: a meta-analysis of randomised controlled trials. Lancet Neurol.

[71] Montero-Odasso M, van der Velde N, Martin FC, Petrovic M, Tan MP, Ryg J (2022). World guidelines for falls prevention and management for older adults: a global initiative. Age Ageing.

[72] Jackson S, Ham RJ, Wilkinson D (2004). The safety and tolerability of donepezil in patients with Alzheimer’s disease. Br J Clin Pharmacol.

[73] Larsson V, Aarsland D, Ballard C, Minthon L, Londos E (2010). The effect of memantine on sleep behaviour in dementia with Lewy bodies and Parkinson’s disease dementia. Int J Geriatr Psychiatry.

[74] Bullock R, Touchon J, Bergman H, Gambina G, He Y, Rapatz G (2005). Rivastigmine and donepezil treatment in moderate to moderately-severe Alzheimer’s disease over a 2-year period. Curr Med Res Opin.

[75] Blain H, Gamon L, Aliaga B, Soriteau L, Raffort N, Miot S (2021). Self-reported fatigue: a significant risk factor for falling in older women and men. Exp Gerontol.

[76] Renner SW, Cauley JA, Brown PJ, Boudreau RM, Bear TM, Blackwell T (2021). Higher fatigue prospectively increases the risk of falls in older men. Innov Aging.

[77] Pandis D, Scarmeas N (2012). Seizures in Alzheimer disease: clinical and epidemiological data. Epilepsy Curr.

[78] Friedman D, Honig LS, Scarmeas N (2012). Seizures and epilepsy in Alzheimer’s disease. CNS Neurosci Ther.

[79] Eddy CM, Rickards HE, Cavanna AE (2011). The cognitive impact of antiepileptic drugs. Ther Adv Neurol Disord.

[80] Cerejeira J, Lagarto L, Mukaetova-Ladinska EB (2012). Behavioral and psychological symptoms of dementia. Front Neurol.

[81] Pariente A, Sanctussy DJR, Miremont-Salamé G, Moore N, Haramburu F, Fourrier-Réglat A (2010). Factors associated with serious adverse reactions to cholinesterase inhibitors: a study of spontaneous reporting. CNS Drugs.

[82] Wynn ZJ, Cummings JL (2004). Cholinesterase inhibitor therapies and neuropsychiatric manifestations of Alzheimer’s disease. Dement Geriatr Cogn Disord.

[83] Van Laar T, De Deyn PP, Aarsland D, Barone P, Galvin JE (2011). Effects of cholinesterase inhibitors in Parkinson’s disease dementia: a review of clinical data. CNS Neurosci Ther.

[84] Poewe W (2008). When a Parkinson’s disease patient starts to hallucinate. Pract Neurol.

[85] Villarejo A, Camacho A, García-Ramos R, Moreno T, Penas M, Juntas R (2003). Cholinergic-dopaminergic imbalance in Pisa syndrome. Clin Neuropharmacol.

[86] Starr JM (2007). Cholinesterase inhibitor treatment and urinary incontinence in Alzheimer’s disease. J Am Geriatr Soc.

[87] Reeve E, Moriarty F, Nahas R, Turner JP, Kouladjian O’Donnell L, Hilmer SN (2017). A narrative review of the safety concerns of deprescribing in older adults and strategies to mitigate potential harms. Expert Opin Drug Saf.

[88] Doody RS, Geldmacher DS, Gordon B, Perdomo CA, Pratt RD (2001). Open-label, multicenter, phase 3 extension study of the safety and efficacy of donepezil in patients with Alzheimer disease. Arch Neurol.

[89] Roitto HM, Kautiainen H, Öhman H, Savikko N, Strandberg TE, Raivio M (2018). Relationship of neuropsychiatric symptoms with falls in Alzheimer’s disease—does exercise modify the risk?. J Am Geriatr Soc.

[90] Roitto HM, Öhman H, Salminen K, Kautiainen H, Laurila J, Pitkälä KH (2020). Neuropsychiatric symptoms as predictors of falls in long-term care residents with cognitive impairment. J Am Med Dir Assoc.

[91] Perri G-A, Liao P (2018). A palliative care perspective on deprescribing cholinesterase inhibitors in Alzheimer’s disease. Curr Med Res Opin.

[92] Kwak YT, Han I-W, Suk S-H, Koo M-S (2009). Two cases of discontinuation syndrome following cessation of memantine. Geriatr Gerontol Int.

[93] Weisbrod N (2022). Primary palliative care in dementia. Neurotherapeutics.

[94] Niznik JD, Zhao X, He M, Aspinall SL, Hanlon JT, Hanson LC (2020). Risk for health events after deprescribing acetylcholinesterase inhibitors in nursing home residents with severe dementia. J Am Geriatr Soc.

[95] Moo LR, Martinez E, Padala K, Dunay MA, Scali RR, Chen S (2021). Unexpected findings during double-blind discontinuation of acetylcholinesterase inhibitor medications. Clin Ther.

[96] Renn BN, Asghar-Ali AA, Thielke S, Catic A, Martini SR, Mitchell BG (2018). A systematic review of practice guidelines and recommendations for discontinuation of cholinesterase inhibitors in dementia. Am J Geriatr Psychiatry.

[97] Neumann S, Taylor J, Bamford A, Metcalfe C, Gaunt DM, Whone A (2021). Cholinesterase inhibitor to prevent falls in Parkinson’s disease (CHIEF-PD) trial: a phase 3 randomised, double-blind placebo-controlled trial of rivastigmine to prevent falls in Parkinson’s disease. BMC Neurol.

